# A Case of Intravascular Myopericytoma: A Painful Subcutaneous Tumor With an Intraosseous Lesion

**DOI:** 10.7759/cureus.72695

**Published:** 2024-10-30

**Authors:** Chikako Sato, Oh Takahashi, Sho Ogata, Mai Fujisaku, Hiromi Edo, Kimiya Sato, Michiro Susa, Susumu Matsukuma

**Affiliations:** 1 Department of Laboratory Medicine, National Defense Medical College Hospital, Saitama, JPN; 2 Department of Pathology and Laboratory Medicine, National Defense Medical College, Saitama, JPN; 3 Department of Radiology, National Defense Medical College Hospital, Saitama, JPN; 4 Department of Basic Pathology, National Defense Medical College, Saitama, JPN; 5 Department of Orthopedic Surgery, National Defense Medical College Hospital, Saitama, JPN

**Keywords:** angioleiomyoma, intravascular myopericytoma, myopericytoma, myopericytomatosis, pericytoma

## Abstract

Myopericytoma is a rare perivascular myoid neoplasm that typically arises in the dermis or subcutaneous tissue, with an intravascular variant also reported. We present a case of an elderly man with a subcutaneous nodule in his lower leg that had persisted for four decades, accompanied by pain in the last several years. Imaging revealed a 20-mm elongated subcutaneous nodule along with a similar intraosseous lesion in the ipsilateral fibula. Histologically, the subcutaneous lesion appeared as a multinodular, vascular-rich tumor, characterized by myoid spindle cells arranged concentrically around vessels of varying sizes. These myoid cells were immunoreactive for smooth muscle markers. Additionally, the tumor exhibited both a feeding artery and a draining vein, mimicking a vascular malformation. Another portion of the subcutaneous nodule was surrounded by a venous-type vascular structure. These findings supported a diagnosis of intravascular myopericytoma, with its extension possibly being myopericytomatosis.

## Introduction

Myopericytoma is a rare perivascular myoid neoplasm that typically involves the dermis or subcutaneous tissue of distal extremities [1,2]. It usually presents as a painless, slow-growing, solitary mass [1,2]. Although most myopericytomas are benign, a few malignant cases have been reported [3,4]. Additionally, rare cases exhibiting diffuse dermal or subcutaneous involvement by microscopic tumor nodules have been described, termed “myopericytomatosis” [5].

Intravascular myopericytoma, an extremely rare variant, is characterized by tumor nodules distributed within the lumen of a vessel. Since MacMenamin et al. first reported a case in 2002, only 19 cases of intravascular myopericytoma have been documented in the English-language literature [4,6-19]. Herein, we report another case of intravascular myopericytoma, detailing its clinicopathological findings.

## Case presentation

**Figure 1:**
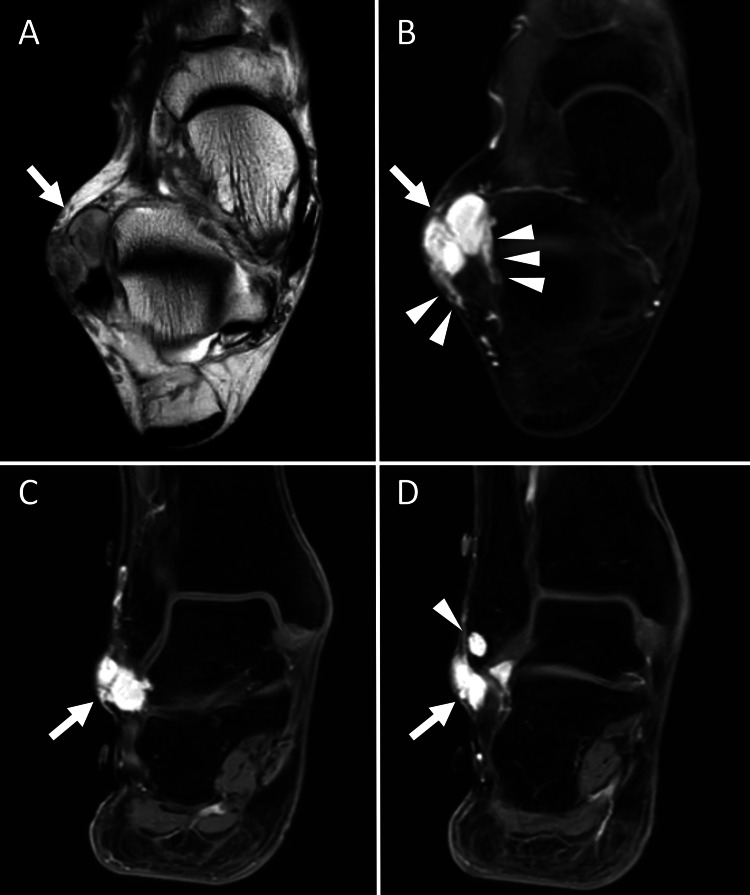
Magnetic resonance imaging findings. Axial T2-weighted image showed a well-defined, mildly hyperintense lesion in the subcutaneous tissue (A, arrow), and contrast-enhanced fat-suppressed T1-weighted MR image demonstrated marked and homogeneous enhancement of the lesion (B, arrow). Dilated blood vessels were observed around the lesion (B, arrowheads).  Coronal contrast-enhanced fat-suppressed T1-weighted MR images also revealed a prominently enhanced subcutaneous lesion over the right lateral malleolus (C, D, arrows), and a similar intraosseous lesion was found at the distal end of the fibula (D, arrowhead).

A 72-year-old man presented with a painful subcutaneous nodule with tenderness over the right lateral malleolus. The nodule had first been noticed by the patient approximately 40 years earlier, and the pain had developed and worsened over the past several years. Laboratory findings were unremarkable. Magnetic resonance imaging (MRI) revealed a well-defined, elongated lesion measuring 20 mm within the subcutaneous tissue, demonstrating mild hyperintensity on T2-weighted images (Figure 1A) and marked enhancement with contrast (Figure 1B, C). Dilated blood vessels were also observed surrounding the lesion (Figure 1B). Additionally, an intraosseous lesion with similar MRI features was identified in the right fibula (Figure 1D). These imaging findings suggested that the subcutaneous nodule was a vessel-rich benign tumor, with differential diagnoses, including glomus tumor, hemangioma, or neurilemmoma. A simple excision of the subcutaneous nodule was performed, while a similar component within the fibula was left untreated. One year after surgery, the patient reported that the pain had subsided, and the residual fibular lesion had not enlarged. At the final follow-up, one year postoperatively, the patient remains alive and well, with no evidence of metastases.

**Figure 2:**
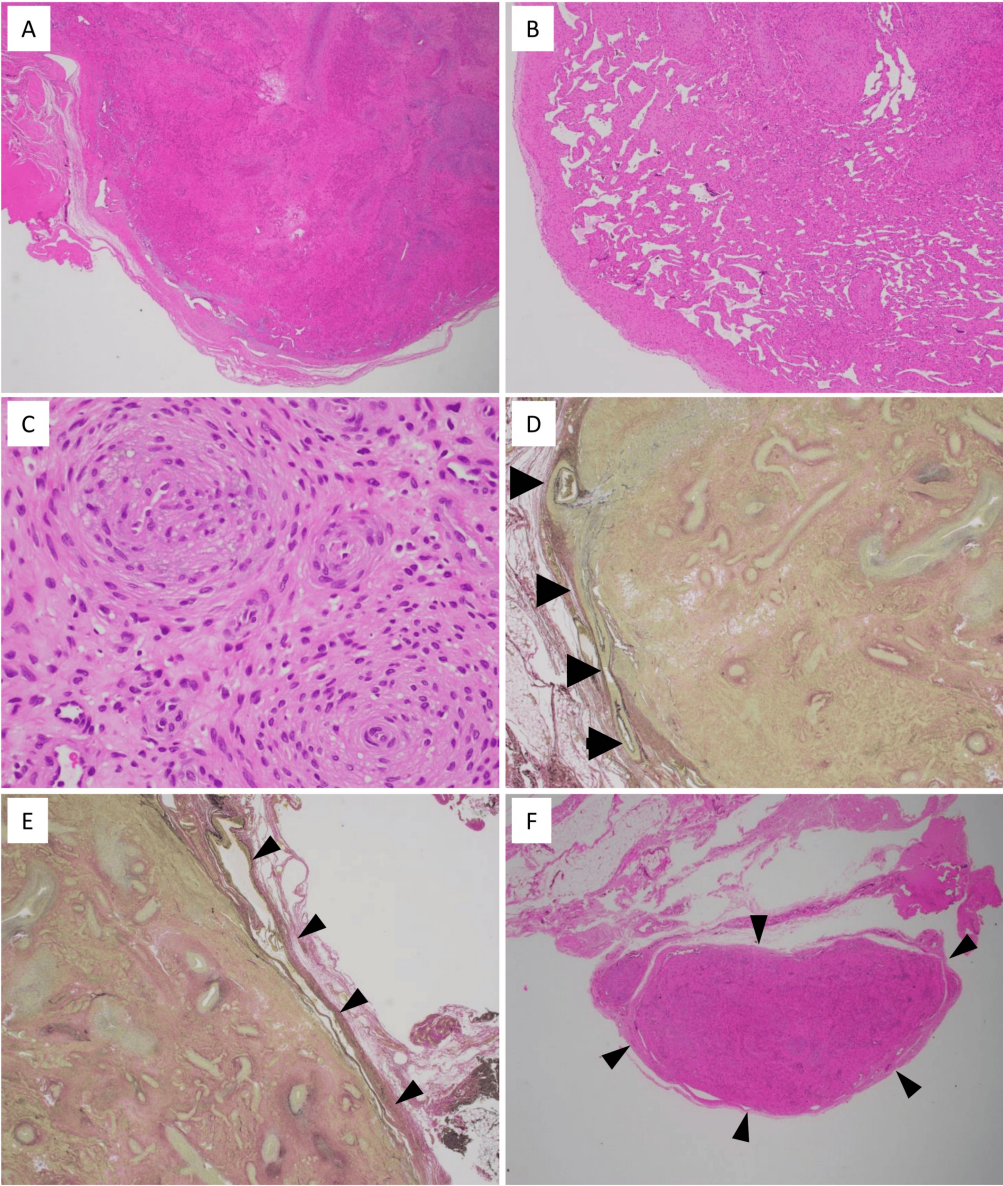
Histology of the subcutaneous lesion. The tumor was well-circumscribed and solid (A), and a hemangiopericytomatous pattern was seen in part (B). Smooth muscle-like plump spindle cells proliferated in a multilayered, concentric perivascular proliferation around various-sized small vessels (C). Neither nuclear atypia nor mitotic figures were observed. An artery (D, arrowheads) and a vein (E, arrowheads) were involved in the tumor. A vein in the fat tissue near the main tumor contained tumor deposit (F, arrowheads).

The removed specimen was divided into two pieces. Histologically, the tumor was well-circumscribed and multinodular. It consisted of smooth muscle-like, plump spindle cells proliferating in a multilayered, concentric growth pattern around small vessels, with hemangiopericytomatous features in some areas (Figure 2A-C). No cellular atypia, tumor necrosis, or mitoses were observed. Immunohistochemically, the proliferating spindle cells were positive for α-smooth muscle actin and h-caldesmon and focally positive for CD34, but negative for desmin and S100 protein. CD31 and ERG were negative in the spindle cells but positive in the endothelial cells lining the vessels. These findings are consistent with typical myopericytoma. Additionally, Elastica van Gieson staining highlighted both an artery and a vein feeding and draining the tumor, and one portion of the tumor was enclosed by a venous vascular structure (Figure 2D, E). These observations suggested that the tumor was an intravascular myopericytoma. An intravascular tumor deposit was also detected within the fat tissue outside the main tumor, indicating possible tumor extension through the adjacent vascular structure (Figure 2F).

## Discussion

We present a rare case of intravascular myopericytoma arising in the right lower leg. Lower extremities are the most frequently reported site for intravascular myopericytoma, followed by the upper extremities, and then the intraorbital area [10], buccal mucosa [7], and trunk [4]. Many were single nodules, with only one being a multinodular lesion [9], and one diffusely involving the venous wall [18]. Ten cases were painful [5,8,9,11-13,15,16,18,19], and four cases were painless [7,10,14,17]. All previous cases were in the subcutaneous or submucosal tissue or dermis. Augstí et al. reported a case of intravascular myopericytoma arising from a cutaneous vascular malformation [14]. The pathogenesis of intravascular myopericytoma remains unclear, but some authors have mentioned an association with a history of venous stasis or minor trauma, as with angioleiomyoma [11]. In the present case, a history of venous stasis or trauma was not evident.

The morphology of myopericytoma is characterized by smooth muscle-like cells proliferating and arranged in a vessel-concentric fashion, features shared to some degree with angioleiomyoma, another tumor in the pericytoma category [1,2]. These tumors share immunohistochemical findings, being positive for α-smooth muscle actin and h-caldesmon, and exhibiting variable results for desmin [1,2,19]. This similarity, and the presence of only subtle morphological differences among these pericytoma-category tumors, is a potential source of confusion between myopericytoma and angioleiomyoma. Furthermore, there is little awareness of this variant, which is reported to be “painful” in contrast to the painless “usual” myopericytoma. The present tumor was vessel-rich and symptomatically painful, and thus might easily have been erroneously considered one of the more frequently experienced painful tumors, such as angioleiomyoma or glomus tumor. Conversely, the “usual” myopericytoma is painless, and so was not a candidate for inclusion in the differential diagnosis among painful subcutaneous nodules. This is a potential pitfall for clinicians, and it is important even for diagnosing pathologists to know that this “painful” (viz. intravascular) variant of myopericytoma exists.

The peculiar feature of the present case was possibly the presence of an intraosseous lesion nearby, something not mentioned in previous reports of intravascular myopericytoma. Unfortunately, the intraosseous lesion had not been confirmed histologically because myopericytomas are themselves considered benign, and the MRI findings of the intraosseous lesion remained unchanged throughout the follow-up period. Most myopericytomas do not recur even if insufficiently excised [1]. However, Mentzel et al. reported two recurrent cases: one was a malignant myopericytoma, but the other was an intravascular variant that had been excised piecemeal [4], and the latter appeared similar to the present case. In the present case, excised specimens of the subcutaneous tumor contained a separate deposit within a vein in the fat tissue. The fibular intraosseous lesion, which was found to be similar on MRI, was considered likely to represent vascular spread from the subcutaneous tumor. The present case might be said to represent “myopericytomatosis” and, as such, to not be easy for total radical resection, thus carrying a risk of becoming a residual or recurrent tumor. This tendency to spread via the vasculature may well be natural for “intravascular” myopericytoma, and for that reason, intravascular variant cases might need to be followed up more closely, especially in cases in which excision was piecemeal.

## Conclusions

We describe a case of subcutaneous intravascular myopericytoma with intraosseous spread, which might be referred to as “myopericytomatosis.” This “painful” variant of myopericytoma might be misdiagnosed as one of the other “painful” pericytoma-category tumors and carries a potential risk of local recurrence. To elucidate the clinicopathological findings and pathogenesis of intravascular myopericytoma more precisely, further case accumulation with correct diagnosis and thorough investigation will be required.
